# Deletion mapping of chromosome 16q in hepatocellular carcinoma

**DOI:** 10.1038/sj.bjc.6690431

**Published:** 1999-05

**Authors:** Z Piao, C Park, J J Kim, H Kim

**Affiliations:** Department of Pathology, Yonsei University, CPO Box 8044, Seoul, Korea

**Keywords:** hepatocellular carcinoma, allelic imbalance, tumour suppressor genes

## Abstract

Hepatocellular carcinoma (HCC) frequently shows an allelic imbalance (AI) on chromosome 16q. In order to define the commonly affected regions on chromosome 16q, we assessed AI studies in 41 HCCs using a panel of 37 microsatellite markers. Thirty-five cases (85%) showed AI at one or more loci. Among the 35 cases with AI, 21 cases showed multiple AI, suggesting the wide scope of deletion on the long arm of chromosome 16, and the remaining 14 cases showed partial AI. Detailed deletion mapping identified two independent commonly deleted regions on this chromosome arm. These included the D16S3106 locus and D16S498 locus. In conclusion, we have demonstrated frequent AI on 16q in HCCs and identified two loci with frequent AI, which may harbour new tumour suppressor genes. © 1999 Cancer Research Campaign


*British Journal of Cancer* (1999) **80**(5/6), 850–854
© 1999 Cancer Research Campaign
Article no. bjoc.1998.0431

### Deletion mapping of chromosome 16q in hepatocellular carcinoma

**Z Piao, C Park, JJ Kim and H Kim**

Department of Pathology, Yonsei University, College of Medicine, CPO Box 8044, Seoul, Korea

**Summary** Hepatocellular carcinoma (HCC) frequently shows an allelic imbalance (AI) on chromosome 16q. In order to define the commonly affected regions on chromosome 16q, we assessed AI studies in 41 HCCs using a panel of 37 microsatellite markers. Thirty-five cases (85%) showed AI at one or more loci. Among the 35 cases with AI, 21 cases showed multiple AI, suggesting the wide scope of deletion on the long arm of chromosome 16, and the remaining 14 cases showed partial AI. Detailed deletion mapping identified two independent commonly deleted regions on this chromosome arm. These included the D16S3106 locus and D16S498 locus. In conclusion, we have demonstrated frequent AI on 16q in HCCs and identified two loci with frequent AI, which may harbour new tumour suppressor genes.

**Keywords:** hepatocellular carcinoma; allelic imbalance; tumour suppressor genes

Hepatocellular carcinoma (HCC) is one of the most common cancers in Asia and Africa (Okuda et al, 1987; Di Bisceglie et al, 1988). However, the molecular mechanism of hepatocarcinogenesis is still unclear. It is generally assumed that the non-random loss of heterozygosity (LOH) detected by restriction fragment length polymorphism (RFLP) markers or allelic imbalance (AI) by using microsatellite markers in a specific region may indicate the existence of a tumour suppressor gene at or near the tested region which is involved in the development or progression of HCC (Fujimori et al, 1991; Weinberg, 1991). AI encompasses LOH and allelic gain; however, cases with marked reduction of intensity of one allele represent LOH rather than allelic gain (Elder et al, 1994). Thus, many chromosomal arms have been reported in HCC as candidate sites for putative tumour suppressor genes, including 1p, 4q, 8p, 13q, 16q and 17p (Tsuda et al, 1990; Fujimori et al, 1991). Among these alterations, LOH on chromosome 16q has been reported to occur more frequently in HCCs of poor differentiation or large size, and with metastasis (Tsuda et al, 1990; Nishida et al, 1992). Recently, in an allelotype study on HCC, we also identified frequent LOH (LOH > 50%) on chromosome 16q, confirming that candidate tumour suppressor genes may be located on this chromosome (Piao et al, 1998).

Deletion and rearrangement of chromosome 16q are also frequently seen in other cancers, including breast cancer, prostate carcinoma and Wilms' tumour (Carter et al, 1990; Maw et al, 1992; Lindblom et al, 1993; Cleton-Jansen et al, 1994; Dorion-Bonnet et al, 1995; Suzuki et al, 1996; Latil et al, 1997). The common deleted regions in these tumours have been identified at 16q22.1 and 16q24-qter (Maw et al, 1992; Dorion-Bonnet et al, 1995; Suzuki et al, 1996). However, it is difficult to ascertain whether LOH reported in different types of cancers is due to the loss of the same tumour suppressor genes in different cancers, or to the loss of distinct genes specific for each tumour type, because different microsatellite or RFLP markers had been used in these studies.

In an effort to define the commonly affected region on chromosome 16q for further positional cloning of the putative tumour suppressor gene in HCC, we performed detailed deletion mapping studies of chromosome 16q in 41 HCCs with 37 microsatellite markers.

*Received 14 April 1998*
*Revised 23 November 1998*
*Accepted 4 December 1998*

*Correspondence to:* H Kim

### MATERIALS AND METHODS

### Tissue selection and DNA extraction

HCCs and adjacent non-tumourous tissues were obtained from 41 patients with HCC. Of these, 39 were obtained from surgical resections performed at Yonsei University College of Medicine, Seoul, Korea, from January 1995 to September 1996. Two HCC tissues were gifts from Dr Uchida, Nihon University School of Medicine, Tokyo, Japan. There were 28 (68.3%) cases positive for HBsAg, two (4.9%) were positive for anti-hepatitis C virus antibody, and the remaining 11 (26.8%) were unrelated to the viral markers. Twenty-four (58.5%) HCCs were detected in cirrhotic livers. Nine cases (22%) were small (≤ 3 cm) and 32 (78%) were advanced (> 3 cm) HCCs (Yumoto et al, 1995). Tumour differentiation was graded according to the criteria of Kanai et al (1987): seven (17%) HCCs were well-differentiated, 23 (56%) were moderately differentiated and 11 (27%) were poorly differentiated according to this grading system. The samples were freshly obtained, immediately frozen in liquid nitrogen and stored at –70°C until analysis. A microdissection technique using a cryostat was used to separate the tumour cells from adjacent normal tissues. Genomic DNA was prepared by the sodium dodecyl sulphate (SDS)–proteinase K and phenol–chloroform extraction method (Gruis et al, 1993).

**Table 1** Allelic imbalance (AI) on 16q in 41 hepatocellular carcinomas

| Locus | Chromosome site | AI/Informative | Percentage |
|---|---|---|---|
| D16S3071 | | 11/26 | 42.3 |
| D16S494 | 16q21 | 12/26 | 46.2 |
| D16S3132 | | 17/33 | 51.5 |
| D16S3143 | | 10/20 | 50.0 |
| D16S514 | 16q21 | 14/26 | 53.9 |
| D16S3129 | | 7/17 | 41.2 |
| D16S503 | 16q21 | 6/20 | 30.0 |
| D16S400 | 16q21 | 7/13 | 53.8 |
| D16S3050 | | 14/31 | 45.2 |
| D16S3067 | | 10/27 | 37.0 |
| D16S3095 | | 9/32 | 28.1 |
| D16S3059 | | 9/26 | 39.1 |
| D16S3106 | | 14/22 | 63.6 |
| D16S3033 | | 11/27 | 40.7 |
| D16S512 | 16q22.1 | 12/35 | 34.3 |
| D16S515 | | 10/34 | 29.4 |
| D16S3097 | | 12/33 | 36.4 |
| D16S3101 | | 9/24 | 37.5 |
| D16S3125 | | 8/19 | 42.1 |
| D16S518 | | 13/29 | 44.8 |
| D16S3029 | | 11/24 | 45.8 |
| D16S3049 | | 9/25 | 36.0 |
| D16S3096 | | 10/24 | 41.7 |
| D16S516 | 16q24.1 | 15/29 | 51.7 |
| D16S3144 | | 5/12 | 41.7 |
| D16S504 | | 10/25 | 40.0 |
| D16S3040 | | 7/19 | 36.8 |
| D16S507 | | 10/20 | 50.0 |
| D16S505 | | 18/36 | 50.0 |
| D16S402 | 16q24.2 | 16/33 | 48.5 |
| D16S3037 | | 14/29 | 48.5 |
| D16S520 | | 12/28 | 42.1 |
| D16S498 | | 22/34 | 64.7 |
| D16S3074 | | 11/29 | 37.9 |
| D16S3048 | | 8/21 | 38.1 |
| D16S3063 | | 5/25 | 20.0 |
| D16S413 | 16q24.3 | 10/26 | 38.5 |

### Analysis of AI using microsatellite markers

A total of 37 microsatellite markers were used, which were obtained from Research Genetics (Huntsville, AL, USA). These are shown in Table 1. Hot-start polymerase chain reactions (PCRs) were performed in a Perkin-Elmer 480 thermal cycler with 20 μl volume containing 1.5 mM magnesium chloride, 20 pmol primer, 0.2 mM each dATP, dGTP, dTTP, 5 μM dCTP, 1 μCi [$\alpha^{32}$P]-dCTP (3000 Ci $mmol^{-1}$; NEN DuPont, Boston, MA, USA), 50 ng sample DNA, 1 × PCR buffer (containing 20 mM Tris–HCl, pH 8.4, 50 mM potassium chloride) and 1.25 U *Taq* polymerase (GIBCO-BRL, Grand Island, NY, USA). DNA amplification was performed with 25 cycles consisting of denaturation at 95°C for 30 s, primer annealing at 55°C for 30 s, and elongation at 72°C for 15 s. PCR products were diluted twofold with stop solution (95% formamide, 20 mM EDTA, 0.05% xylene cyanol and 0.05% bromophenol blue). Three microlitres of mixture were loaded onto 6% denaturing polyacrylamide gel containing 5.6 M urea. The gel was dried and exposed to Kodak XAR-5 film (Kodak, Rochester, NY, USA). AI was scored when band intensity of one allelic marker was significantly decreased (more than 70% reduction) in tumour DNA compared with that in normal DNA (Elder et al, 1994).

### RESULTS

### Defining a minimal region of AI on chromosome 16q

Forty-one HCCs were screened for AI with a panel of 37 microsatellite markers specific for chromosome 16q loci. This made it possible to find the regions showing frequent (> 60%) AI and mapping of the deletion regions on chromosome 16q. The representative autoradiographs of the several markers are shown in Figure 1. All 41 cases were informative at several loci on 16q and 35 cases (85%) showed AI at one or more loci. The overall frequency of deletion at each locus and its linkage ordering based on the Genethon Linkage Map is shown in Table 1 (Dib et al, 1996). Markers D16S498 (64%) showed the highest AI of the markers tested (Table 1). The patterns of AI at several specific loci are shown in Figure 2. Two independent regions of frequent AI were defined: the first region was between D16S3059 and D16S3033, encompassed by approximately a 2 cM region, and defined by the D16S3106 locus; the second region was defined by the D16S498 locus between the D16S520 and D16S3074 loci, and encompassed approximately by a 2 cM region (Figure 2). Several independent regions of relatively frequent (> 40%) AI were also

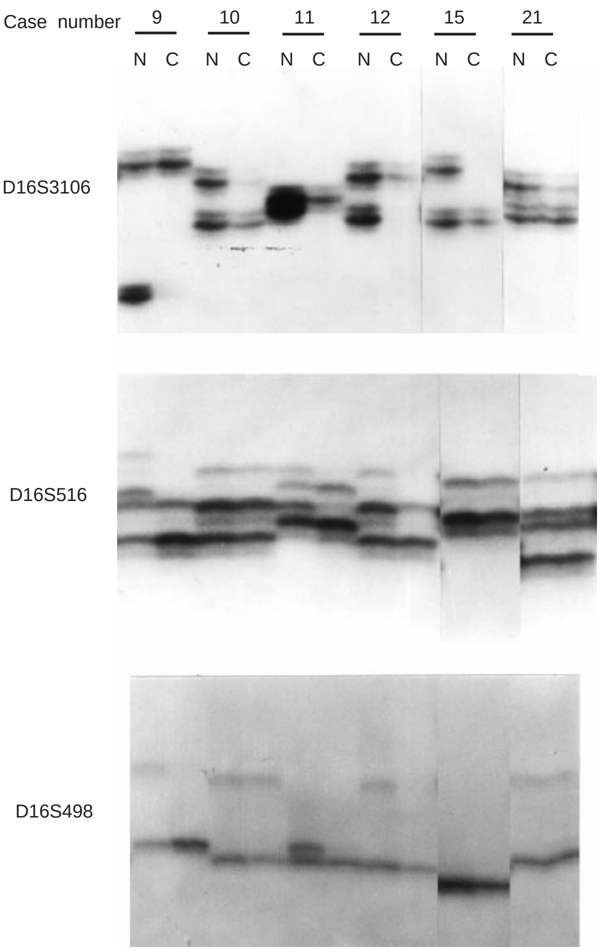

**Figure 1** Representative autoradiographs of allelic imbalance (AI). The carcinoma (C) and corresponding non-tumourous tissue (N) are shown with microsatellite markers indicated at the left. AI is seen in cases 9, 10, 11, 12, 15 and 21due to loss of the lower allele in cases 9, 11 and 12, and oss of the upper allele in cases 10, 15 and 21 with marker D16S3106. AI is seen in casse 9, 11 and 12 with markers of D16S516 and D16S498

© Cancer Research Campaign 1999

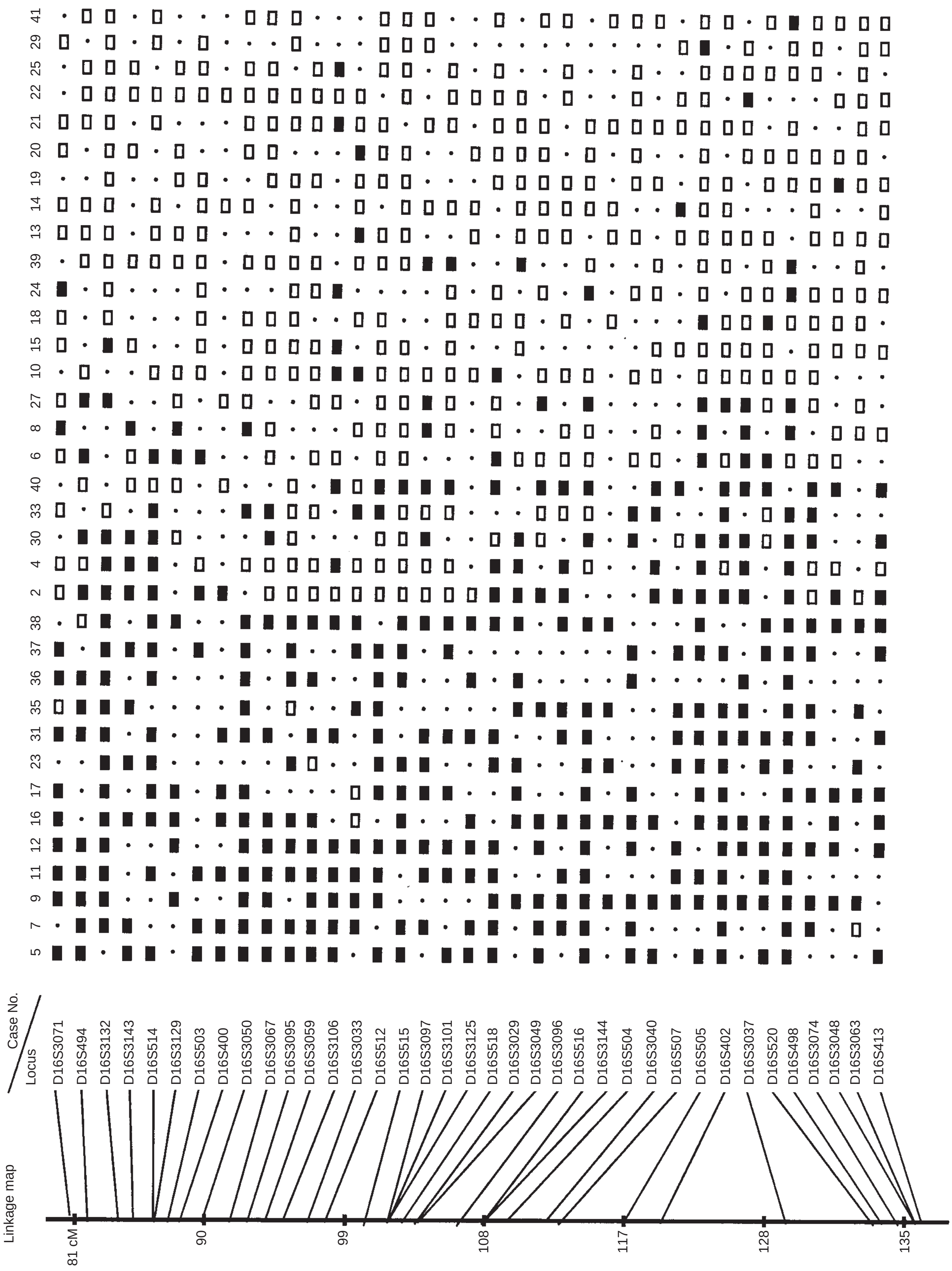

**Figure 2** Schematic representation of allelic imbalance in hepatocellular carcinoma of 37 microsatellite markers mapping from D16S3071 to D16S413. The markers are listed in relative positions from centromeric to the most telomeric. Thirty-five cases show AI at one or more loci according to their linkage order ■ : allelic imbalance; □: retention of heterozygosity; ● : not informative

 *© Cancer Research Campaign 1999*

**Table 2** Comparison of clinicopathologic features of hepatocellular carcinoma according to the alletic imbalance (AI) status of 16q

| | 16q AI status | | | AI of D16S3106 | | AI of D16S498 | |
|---|---|---|---|---|---|---|---|
| Variable Category | Wide-scope | Restricted | Absent | Present | Absent | Present | Absent |
| Tumour size | | | | | | | |
| < 3 cm | 3 | 3 | 3 | 2 | 4 | 4 | 3 |
| > 3 cm | 18 | 11 | 3 | 12 | 4 | 18 | 10 |
| Tumour differentiation | | | | | | | |
| Well | 1 | 4 | 2 | 3 | 3 | 1 | 4 |
| Moderate | 14 | 7 | 2 | 6 | 2 | 15 | 5 |
| Poor | 6 | 3 | 2 | 5 | 3 | 6 | 4 |
| Cirrhosis in adjacent liver | | | | | | | |
| Present | 11 | 10 | 3 | 7 | 6 | 12 | 8 |
| Absent | 10 | 4 | 3 | 7 | 2 | 10 | 5 |
| Serum HBsAg | | | | | | | |
| Present | 13 | 11 | 4 | 11 | 5 | 15 | 7 |
| Absent | 8 | 3 | 2 | 3 | 3 | 7 | 6 |

defined: the first region was between D16S494 and D16S503, encompassed by a 6 cM region, and defined by the D16S3132 and D16S514 loci (Figure 2); the second region was between the D16S503 and D16S3067 loci, encompassed by a 4 cM region, and defined by the D16S400 and D16S3050 loci (Figure 2); the third region was between the D16S3096 and D16S504 loci, encompassed by a 2 cM region and defined by the D16S516 locus (Figure 2); the fourth AI region was defined by the D16S507 and D16S505 loci. Among the 35 HCCs with AI on chromosome 16q, 21 HCCs (cases 2, 4–9, 11, 12, 16, 17, 23, 27, 30, 31, 33, 35–38, 40) showed AI at multiple informative loci, indicating a wide scope or entire deletion on the long arm of chromosome 16 (Figure 2). The remaining 14 HCCs showed deletions involving restricted regions of 16q.

### Association of AI with clinicopathological parameters

In order to examine the relationship between AI on 16q and the clinicopathological findings, we compared the AI of six regions with various clinicopathological data, including HBV infection, cirrhosis, tumour size and tumour differentiation. Most of the HCCs having wide-scope deletion on the 16q had a trend toward large-size tumour formation (18/21, 86%) and less (moderate and poor) differentiation (20/21, 95%); however, there was no significant association between the AI of 16q and clinicopathological parameters (Table 2). HCCs with AI in D16S3106 had a trend toward large-size tumour formation (12/14, 86% vs 4/8, 50%, $P = 0.07$) and HCCs with AI in D16S498 had a tendency of less (moderate and poor) differentiation (21/22, 95% vs 9/13, 69%, $P = 0.073$). However, there was no significant association between the two commonly deleted regions and clinicopathological parameters (Table 2).

### DISCUSSION

Non-random deletion or allelic imbalance (AI) in HCC has been reported on chromosomes 1p, 4q, 8p, 13q, 16q and 17p (Tsuda et al, 1990; Fujimori et al, 1991), indicating the existence of putative tumour suppressor genes on these chromosomes (Weinberg, 1991). Previously, we found that D16S752 was the most commonly affected locus on the long arm of chromosome 16 (Piao et al, 1998). However, this locus can not be places at specific regions on the published linkage map (Dib et al, 1996). Therefore, we screened an area of approximately 55 cM of 16q in 41 HCCs to further narrow down the regions of the markers. The results indicated that there were two independent most frequent AI on the long arm of chromosome 16 with a panel of 37 microsatellite common AI regions on chromosome 16q which both gave a very comprehensive evidence of the presence of several tumour suppressor genes involved in hepatocarcinogenesis on this chromosomal arm.

AI on chromosome 16q has been observed at a high frequency in HCC (Tsuda et al, 1990; Fujimori et al, 1991). Tsuda et al (1990) detected that the common region of allelic loss on chromosome 16 was between the HP locus (16q22.1) and the CTRB locus (16q22.3–q23.2) with RFLP markers. Yeh et al (1996) also reported that the common deleted region was mapped to 16q22–23 in HCC. Frequent AI on chromosome 16q has also been reported in other tumour types including breast cancer (Lindblom et al ,1993; Cleton-Jansen et al, 1994; Dorion-Bonnet et al, 1995), prostate carcinoma (Carter et al, 1990; Latil et al, 1997) and Wilms' tumour (Maw et al, 1992). Two regions on chromosome 16q have been shown to have a very high frequency of AI in breast cancer; one maps to region 16q22.1 and the other to 16q24.2-qter (Cleton-Jansen et al, 1994). Similarly, Suzuki et al (1996) identified three distinct commonly deleted regions which were located at 16q22.1–q22.3, 16q23.2–q24.1 and 16q24.3-qter in prostate cancers. Latil et al (1997) also identified three distinct commonly deleted regions in prostate cancers; at 16q24.3 between markers D16S520 and D16S413, at 16q22.1 defined by markers D16S347 and D16S318, at 16q23.2 between markers D16S518 and D16S507. Recently, Chen et al (1996) defined the minimal deleted region in breast cancer that is most commonly affected as 16q23.3–q24.1, locus D16S518 between markers D16S515 and D16S504, which the other two regions of interest for LOH are located at 16q24.2, locus D16S402 and 16q21, locus D16S400. Specific chromosomal regions showing AI in this study were similar to those reported previously for 16q21–q24, but there were some differences. Our study showed that two independent AI regions (loci) were defined on chromosome 16q in HCC. Among these two regions, the D16S498 region was in agreement with previously described areas in prostate carcinoma; 16q24.3 at loci between D16S520 and D16S413 (Latil et al, 1997). However, we demonstrated that the D16S498 locus shows most frequent AI at

© Cancer Research Campaign 1999

this region by detailed deletion mapping analysis. The D16S3106 locus, to our knowledge, had not been reported previously. We also demonstrated four independent relatively frequent AI regions: the first region, the D16S3132 and D16S514 loci; the second region, the D16S400 and D16S3050 loci; the third region, the D16S516 locus; the fourth region, the D16S507 and D16S505 loci. Among these regions, the second region was in agreement with previously described areas in breast cancer: 16q21 at locus D16S400 (Chen et al, 1996). However, we found that the D16S3050 locus distal to the D16S400 locus also showed a high frequency of AI (45.2%). Therefore, the frequently deleted region was defined by the D16S400 and D16S3050 loci between D16S503 and D16S3067, encompassed by a 4 cM region. The third region was in agreement with previously described areas in prostate carcinomas; 16q24.3 at loci between D16S518 and D16S507. We defined D16S516 as the most commonly deleted locus in these regions using seven microsatellite markers between D16S518 and D16S507. Although D16S518 was reported as the most frequently affected locus between markers D16S515 and D16S504 in breast cancer (Chen et al, 1996), this locus did not show a high frequency of AI in HCC. Instead, with detailed mapping we defined one independent region of AI between markers D16S515 and D16S504, as D16S516 (51.7%).

The association between allelic loss on chromosome 16q and the progression of human HCC has been proposed by several groups (Tsuda et al, 1990; Nishida et al, 1992; Yumoto et al, 1995). Tsuda et al (1990) reported that allelic loss between HP and CTRB loci occurred more frequently in HCCs of poor differentiation and large size, and with metastasis. Similar results were reported in cancer (Lindblom et al, 1993) and prostate cancer (Suzuki et al, 1996). On the other hand, Chen et al (1996) reported that frequent LOH at a preinvasive stage of breast cancer suggests that a candidate tumour suppressor gene on 16q may play an important role in breast carcinogenesis. Our results showed that AI of 16q in HCC was very frequent and many of them showed wide-scope deletion of the one chromosomal arm. Although we could not find any statistically significant relationship between AI of 16q and clinicopathological parameters, HCCs with AI of 16q had a trend toward large mass formation and poor differentiation. These findings suggest that AI of 16q in HCCs might be related to the HCC progression.

In conclusion, this study demonstrated that chromosome 16q is highly affected by AI in HCC. We narrowed down the location of the minimal deletion regions which are involved in two independent regions (loci) on chromosome 16q.

### ACKNOWLEDGEMENTS

This work was supported by Yonsei University Cancer Research Institute Grant (96–4). The authors would like to thank Dr Uchida, Nihon University School of Medicine, Tokyo, Japan for generously supplying tissues from two HCC cases. We also thank Mr B Ross for assistance with English and Miss EY Kim for photographic assistance.

### REFERENCES

Carter BS, Ewing CM, Ward WS, Treiger BF, Aalders TW, Schalken JA, Epstein JI and Issac WB (1990) Allelic loss of chromosome 16q and 10q in human prostate cancer. *Proc Natl Acad Sci USA* **87**: 8751–8755

Chen T, Sahin A and Aldaz CM (1996) Deletion map of chromosome 16q in ductal carcinoma in situ of the breast: refining a putative tumor suppressor gene region. *Cancer Res* **56**: 5605–5609

Cleton-Jansen AM, Moerland EW, Kuipers Dijkshoorn NJ, Callen DF, Southerland GR, Hansen B, Devilee P and Cornelisse CJ (1994) At least two different regions are involved in allelic imbalance on chromosome arm 16q in breast cancer. *Genes Chromosomes Cancer* **9**: 101–107

Di Bisceglie AM, Rustgi VK, Hoofnagle JH, Dusheiko GM and Lotze MT (1988) Hepatocellular carcinoma. *Ann Intern Med* **108**: 390–401

Dib C, Faure S, Fizames C, Samson D, Drouot N, Vignal A, Millasseau P, Marc S, Hazan J, Seboun E, Lathrop M, Gyapay G, Morissette J and Weissenbach J (1996) A comprehensive genetic map of the human genome based on 5264 microsatellites. *Nature* **380**: 152–154

Dorion-Bonnet F, Mautalen S, Hostein I and Longy M (1995) Allelic imbalance study of 16q in human primary breast carcinomas using microsatellite markers. *Genes Chromosomes Cancer* **14**: 171–181

Elder PA, Bell SM and Knowles MA (1994) Deletion of two regions on chromosome 4 in bladder carcinoma: definition of a critical 750 kB region at 4p16.3. *Oncogene* **9**: 3433–3436

Fujimori M, Tokino T, Hino O, Kitagawa T, Imamura T, Okamoto E, Mitsunobu M, Ishikawa T, Nakagama H, Harada H, Yagura M, Matsubara K and Nakamura Y (1991) Allelotype study of primary hepatocellular carcinoma. *Cancer Res* **51**: 89–93

Gruis NA, Abeln ECA, Bardoel AFJ, Devilee P, Frants RR and Cornelisse CJ (1993) PCR-based microsatellite polymorphisms in the detection of loss of heterozygosity in fresh and archival tumor tissue. *Br J Cancer* **68**: 308–313

Kanai T, Hirohashi S, Upton MP, Noguchi M, Kishi K, Makuuchi M, Yamasaki S, Hasegawa H, Takayasu K, Moriyama N and Shimosato Y (1987) Pathology of small hepatocellular carcinoma. A proposal for new classification. *Cancer* **60**: 810–819

Latil A, Cussenot O, Fournier G, Driouch K and Lidereau R (1997) Loss of heterozygosity at chromosome 16q in prostate adenocarcinoma: identification of three independent regions. *Cancer Res* **57**: 1058–1062

Lindblom A, Rotstein S, Skoog L, Nordenskjold M and Larsson C (1993) Deletion on chromosome 16 in primary familial breast carcinomas are associated with development of distant metastases. *Cancer Res* **53**: 3707–3711

Maw MA, Grundy PE, Millow LJ, Eccles MR, Eccles MR, Dunn RS, Smith PJ, Feinberg AP, Law DJ, Paterson MC, Paterson MC, Telzerow PE, Callen DF, Thompson AD, Richards RI and Reeve AE (1992) A third Wilms' tumor locus on chromosome 16q. *Cancer Res* **52**: 3094–3098

Nishida N, Fukuda Y, Kokuryu H, Sadamoto T, Isowa G, Honda K, Yamaoka Y, Ikenaga M, Imura H and Ishizaki K (1992) Accumulation of allelic loss on arms of chromosomes 13q, 16q and 17q in the advanced stages of human hepatocellular carcinoma. *Int J Cancer* **51**: 862–868

Okuda K, Fujimoto I, Hanai A and Urano Y (1987) Changing incidence of hepatocellular carcinoma in Japan. *Cancer Res* **47**: 4967–4972

Piao Z, Park C, Park JH and Kim H (1998) Allelotype analysis of hepatocellular carcinoma. *Int J Cancer* **75**: 29–33

Suzuki H, Komiya A, Emi M, Kuramochi H, Shiraishi T, Yatani R and Shimazaki J (1996) Three distinct commonly deleted regions of chromosome arm 16q in human primary and metastatic prostate cancers. *Genes Chromosomes Cancer* **17**: 225–233

Tsuda H, Zhang W, Shimosato Y, Yokota J, Terada M, Sugimura T, Miyamura T and Hirohashi S (1990) Allele loss on chromosome 16 associated with progression of human hepatocellular carcinoma. *Proc Natl Acad Sci USA* **87**: 6791–6794

Weinberg RA (1991) Tumor suppressor genes. *Science* **254**: 1138–1146

Yeh SH, Chen PJ, Lai MY and Chen DS (1996) Allelic loss on chromosomes 4q and 16q in hepatocellular carcinoma. *Gastroenterology* **110**: 184–192

Yumoto Y, Hanafusa T, Hada H, Morita T, Ooguchi S, Shinji N, Mitani T, Hamaya K, Koide N and Tsuji T (1995) Loss of heterozygosity and analysis of mutation of p53 in hepatocellular carcinoma. *J Gastroenterol Hepatol* **10**: 179–185

 *© Cancer Research Campaign 1999*
